# Supplementary material for: Thermal Processing of Liquid Egg Yolks Modulates Physio-Chemical Properties of Mayonnaise
Source: Foods. 2022 May 14;11(10):1426. doi: 10.3390/foods11101426 (PMC9141538; doi:10.3390/foods11101426)
Supplement: Supplementary file 1 [file foods-11-01426-s001.zip › foods-1716496-supplementary.pdf]

foods

Supplementary data

Thermal Processing of Liquid Egg Yolks Modulates Physio-Chemical Properties of Mayonnaise

Jou-Hsuan Ho [1], Tan-Ang Lee [1], Nobuaki Namai [2], Shunji Sakai [2], Siao-Syuan Lou [1], Akihiro Handa [3,†] and Wan-Teng Lin [4,*,†]

[1] Department of Food Science, Tunghai University, Taichung 40704, Taiwan; jhho@thu.edu.tw (J.-H.H.); danyanlee@gmail.com (T.-A.L.); cc5cc57788@gmail.com (S.-S.L.)
[2] R&D Division, Kewpie Corporation, Tokyo 182-0002, Japan; nobuaki_namai@kewpie.co.jp (N.N.); shunji_sakai@kewpie.co.jp (S.S.)
[3] Division of Life Science and Engineering, School of Science and Engineering, Tokyo Denki University, Saitama 350-0394, Japan; ahanda@mail.dendai.ac.jp
[4] Department of Hospitality Management, College of Agriculture, Tunghai University, Taichung 40704, Taiwan
* Correspondence: 040770@thu.edu.tw; Tel.: +886-4-2359-0121 (ext. 37709)
† These authors contributed equally to this work.

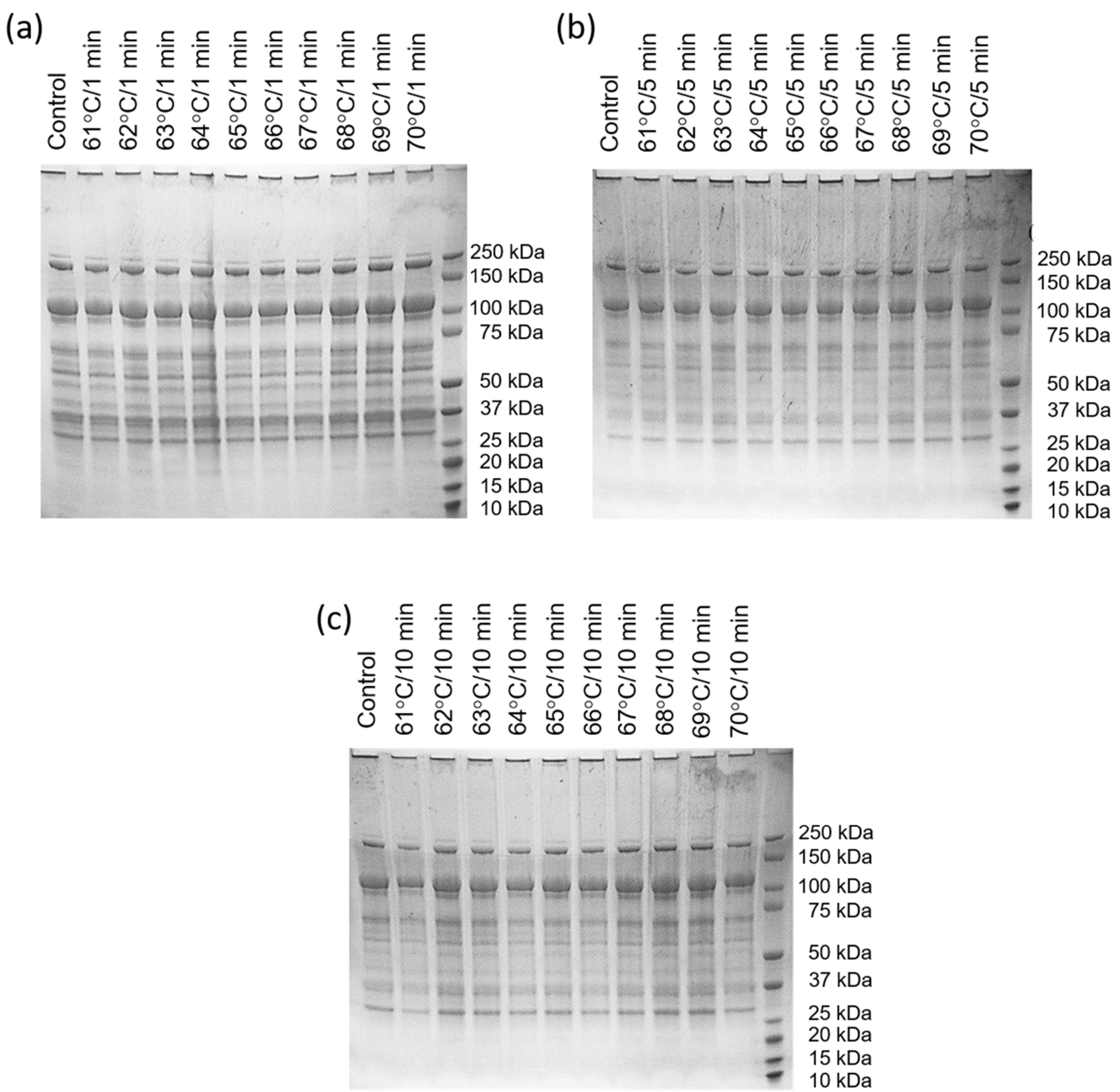

Figure S1. Effect of thermal processing on LEY protein denaturation determined by western blotting.

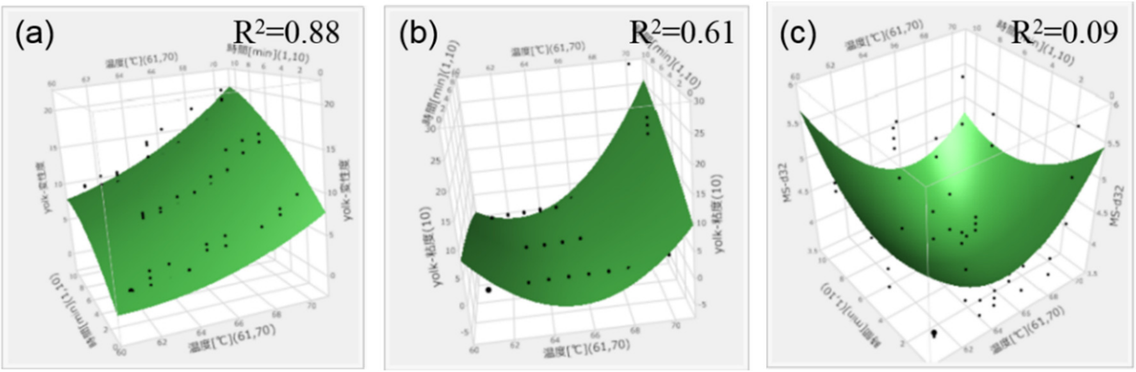

Figure S2. Response surface pattern analysis of LEY with different temperatures and times. (a) Degree of protein denaturation. (b) Viscosity. (c) Particle size.

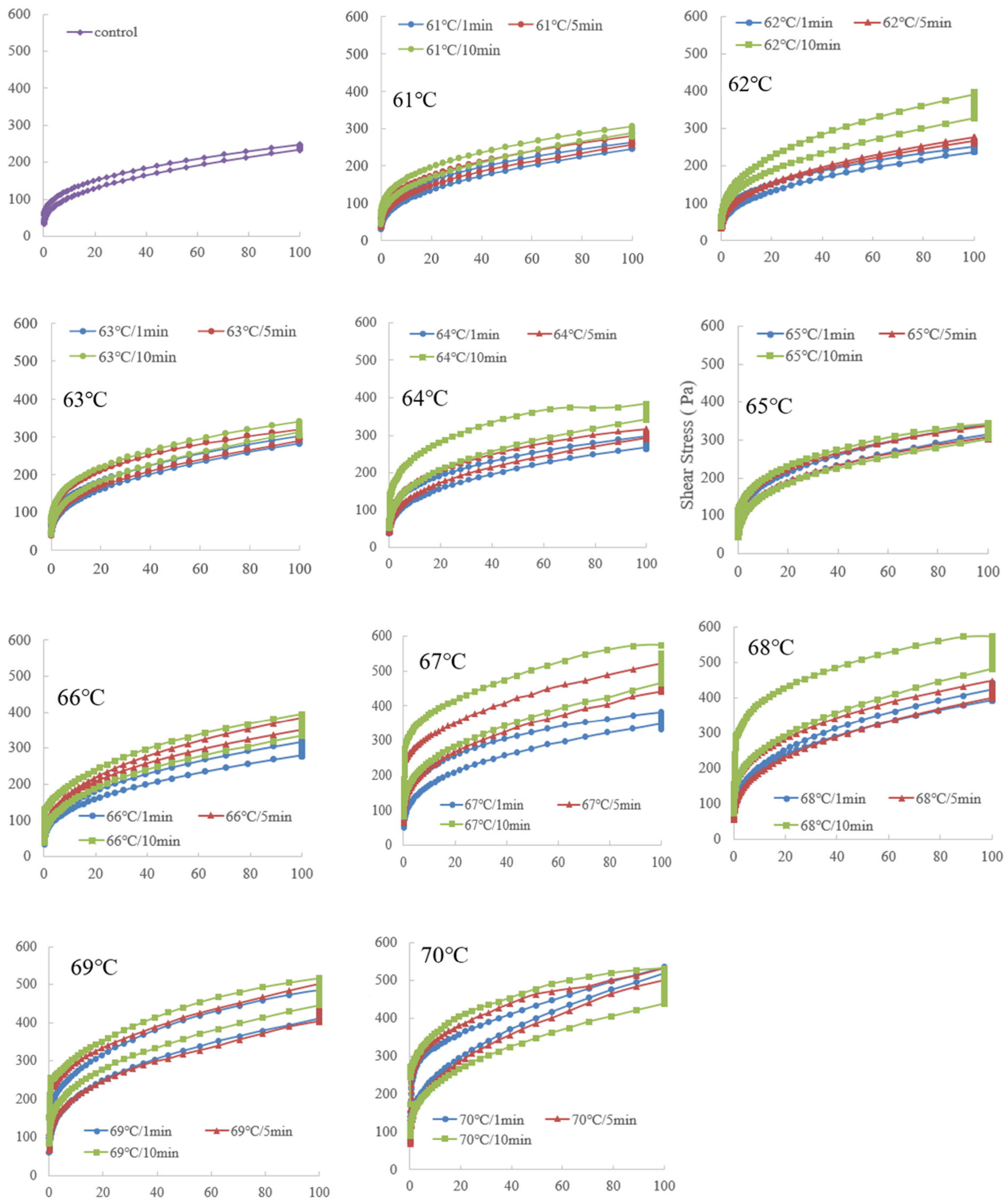

Figure S3. Effect of thermal processing on emulsion property of mayonnaise.

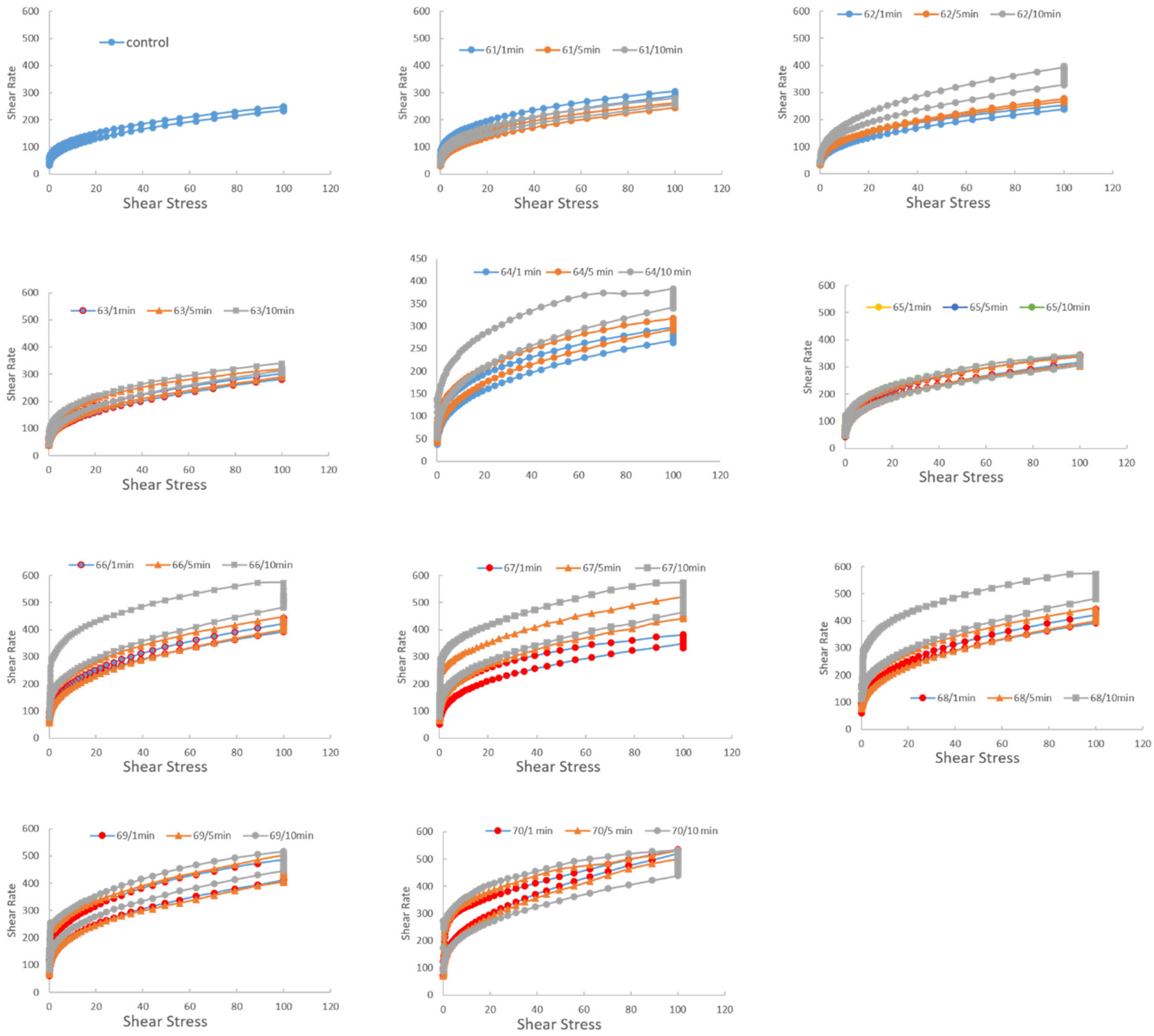

Figure S4. Effect of thermal processing on mayonnaise rheological properties.

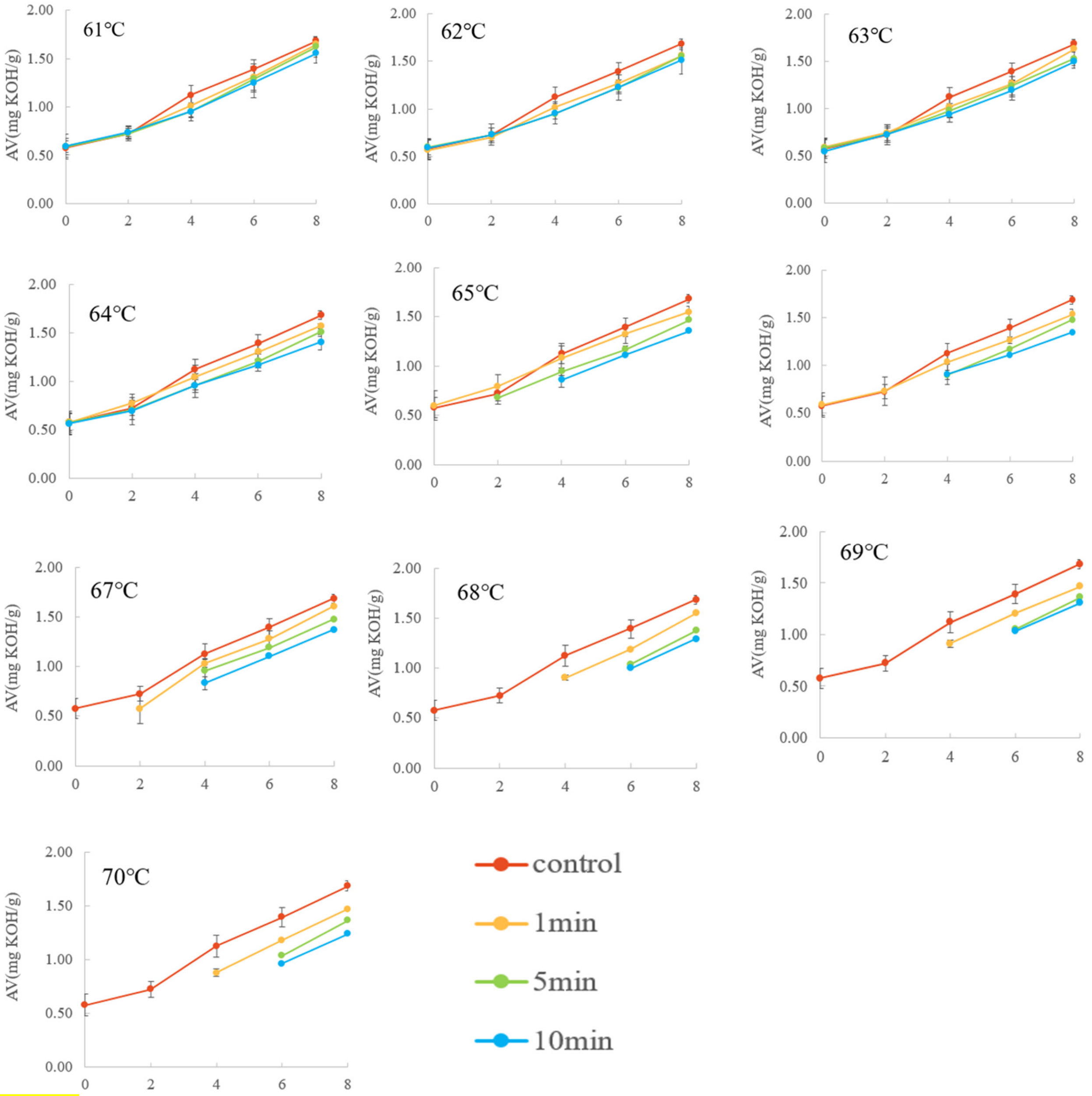

Figure S5: Effect of thermal processing on storage properties (acid values) of mayonnaise.

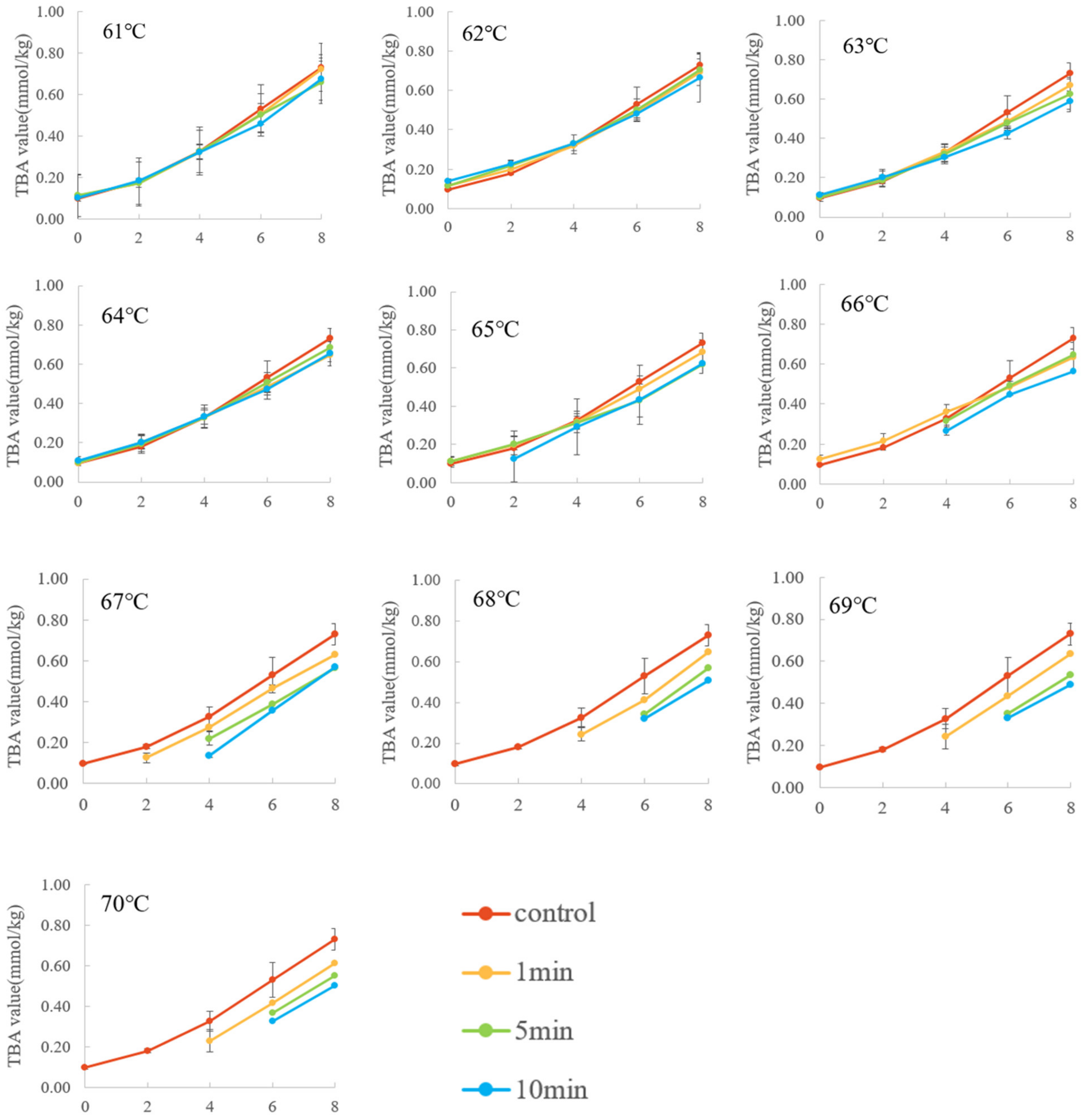

Figure S6: Effect of thermal processing on storage properties (TBA values) of mayonnaise.
